# Clinicopathological Characteristics of Superficial Type Colorectal Adenomas Obtained by Endoscopic Resection

**DOI:** 10.1155/DTE.2.99

**Published:** 1995

**Authors:** Sumio Fujinuma, Yoshihiro Sakai

**Affiliations:** 1 Division of Digestive Endoscopy Ohashi Hospital Toho University School of Medicine 2-17-6 Ohashi, Meguro-ku Tokyo 153 Japan

## Abstract

Colorectal adenomas may be either protruding type or superficial type lesions. To delineate the clinicopathological characteristics of the latter, 153 superficial type adenomas (including the surrounding mucosa) obtained by endoscopic resection were studied morphologically. Superficial type adenomas were defined as flat or flat depressed adenomas with a height of ≤3 mm; histologically, the tubules proliferated horizontally without vertical overlap. The location of tubules in the mucosa was classified as: involvement of the surface layer only (m_1_), deeper invasion not reaching the muscularis mucosae (m_2_), or invasion to the muscularis mucosae (m_3_). The results of analysis indicated: 1) there was no relationship between atypia and size; 2) although macroscopic features (depression, etc.) were associated with the grade of atypia, a closer association was obtained for the location in the mucosa; 3) based on our classification system for tubule location, (m_2_) and (m_3_) adenomas had a significantly higher frequency of depressed type lesions than did m_1_ lesions; and 4) the height of superficial type adenomas was 295 to 413 μm. Height was lowest in the m_3_ group followed by, in ascending order, the m_2_ and m_1_ groups. These morphological and histological characteristics are expected to contribute to improved diagnosis of superficial type adenomas.


*Diagnostic and Therapeutic Endoscopy*, 1995, Vol. 2, pp. 99–105
Reprints available directly from the publisher
Photocopying permitted by license only

© 1995 Harwood Academic Publishers GmbH
Printed in Singapore

### Clinicopathological Characteristics of Superficial Type Colorectal Adenomas Obtained by Endoscopic Resection

SUMIO FUJINUMA and YOSHIHIRO SAKAI

*Division of Digestive Endoscopy, Ohashi Hospital, Toho University School of Medicine, Tokyo, Japan*

*(Received February 27, 1995; in final form, April 4, 1995)*

Colorectal adenomas may be either protruding type or superficial type lesions. To delineate the clinicopathological characteristics of the latter, 153 superficial type adenomas (including the surrounding mucosa) obtained by endoscopic resection were studied morphologically. Superficial type adenomas were defined as flat or flat depressed adenomas with a height of ≤3 mm; histologically, the tubules proliferated horizontally without vertical overlap. The location of tubules in the mucosa was classified as: involvement of the surface layer only ($m_1$), deeper invasion not reaching the muscularis mucosae ($m_2$), or invasion to the muscularis mucosae ($m_3$). The results of analysis indicated: 1) there was no relationship between atypia and size; 2) although macroscopic features (depression, etc.) were associated with the grade of atypia, a closer association was obtained for the location in the mucosa; 3) based on our classification system for tubule location, $m_2$ and $m_3$ adenomas had a significantly higher frequency of depressed type lesions than did $m_1$ lesions; and 4) the height of superficial type adenomas was 295 to 413 µm. Height was lowest in the $m_3$ group followed by, in ascending order, the $m_2$ and $m_1$ groups. These morphological and histological characteristics are expected to contribute to improved diagnosis of superficial type adenomas.

**KEY WORDS:** Adenoma, colon, endoscopic resection, superficial type, rectum

### INTRODUCTION

Previous studies on colorectal adenomas have focused primarily on protruding type lesions (1,2). The high frequency of carcinomas in adenomas among these lesions has provided evidence for the adenoma-carcinoma sequence theory (3,4). Recently, however, increased discovery of flat and depressed type early carcinomas has highlighted the clinical importance of superficial type neoplasms of the colon and rectum, and previously accepted theories on the histogenesis of adenomas are now being reassessed. For protruding type adenomas, lesion size and morphology (pedunculated, spherical, etc.) are apparently related to the probability of coexisting carcinoma. This and other types of evidence have formed the basis for the adenoma-carcinoma sequence theory. However, interest in the *de novo* theory of carcinogenesis has recently been revived by the increased detection of superficial type neoplasms.

To better understand the clinicopathological features of colorectal adenomas, we studied the morphological and histological characteristics of superficial type adenomas that were obtained by endoscopic resection (ER) after injection of physiological saline solution. Clinical materials obtained by ER were used because this technique allows the surrounding mucosa to be sampled at the same time and under the same conditions as the target lesion, a situation that is a prerequisite to a meaningful analysis of superficial-type adenomas.

### SUBJECTS AND METHODS

One hundred fifty-three lesions satisfying the histological criteria for superficial type adenomas were studied. These lesions were identified from among the 466 lesions obtained by ER in this department during the 4.5 years be-

Address for correspondence: Sumio Fujinuma, M.D., Division of Digestive Endoscopy, Ohashi Hospital, Toho University School of Medicine, 2-17-6 Ohashi, Meguro-ku, Tokyo 153, Japan.

tween May 1989 and October 1993. Superficial type adenomas were defined as flat or flat depressed lesions with a height of ≤3 mm. Histologically, the tubules proliferated horizontally without vertical overlap.

Of the 153 lesions studied, 111 were obtained from men (mean age 62.5 years) and 42 from women (mean age 61.9 years). These patients underwent colonoscopic examination because of a positive occult blood reaction or the presence of symptoms such as abdominal pain, constipation, and diarrhea. No patient had previously undergone resection of cancer. Based on gross morphology, these superficial type lesions were classified according to the classification for early gastric cancer as IIa (superficial elevated type, lesions that are slightly higher than the surface of the mucosa), IIb (superficial flat type, lesions with virtually no difference in height from the surrounding mucosa), or IIc (superficial depressed type, lesions that are slightly lower than the surrounding mucosa). Protruding lesions that had a central depression were classified as IIaIIc. Gross lesion morphology was evaluated by referring to endoscopic findings before resection, resected specimens after they were spread out and fixed in formalin, and tissue sections prepared by conventional techniques. Any disagreement in classification was resolved by referring to the tissue sections.

Endoscopically resected materials were gently spread out, pinned down, fixed in 10% formalin, and sliced at 3-mm intervals, taking care to include the largest sectional area of the lesion. The sections were then embedded in paraffin, sliced at a thickness of 4 μm, and stained with hematoxylin-eosin. Histologically, the lesion was examined at low magnification, and location was classified as one of three levels: 1) invasion of the surface layer of the mucosa only ($m_1$, Fig. 1A), 2) deeper invasion but not reaching the muscularis mucosae ($m_2$, Fig. 2A), and 3) invasion to the muscularis mucosae ($m_3$, Fig. 3A). Lesions showing $m_1$ tubules at the periphery and $m_3$ near the center were classified as $m_2$. Endoscopic photographs of the corresponding $m_1$, $m_2$, and $m_3$ lesions are shown in Figures 1B, 2B, and 3B, respectively. According to this classification system, $m_1$ lesions were relatively thin adenomas, while $m_3$ lesions were thick adenomas that occupied all cell layers of the mucosa.

The heights of the lesions and surrounding mucosa were measured from the muscularis mucosae. Lesion thickness was expressed as the mean of the maximum thickness and the minimum thickness measured from the muscularis mucosae. Lesion height was calculated by subtracting the thickness of the surrounding mucosa from lesion thickness (Fig. 4).

Lesions were graded as having mild, moderate, or severe atypia, and their characteristics were compared clinicopathologically. The significance of intergroup differences was tested with the $t$ test and the $\chi^2$ test. Differences associated with $P < 0.05$ were regarded as significant.

### RESULTS

### Adenoma Atypia and Size

Among the 153 superficial type adenomas, the grade of atypia was classified as mild for 9 lesions, moderate for 119 lesions, and severe for 25 lesions. Lesion size was 5.1 ± 1.9 mm (range 3 to 8 mm), 4.9 ± 2.0 mm (range 3 to 11 mm), and 5.5 ± 2.3 mm (range 2 to 9 mm) in the mild, moderate, and severe atypia groups, respectively. These differences were not significant (Table 1).

### Adenoma Atypia and Site

The majority of lesions were situated from the sigmoid colon to the transverse colon. There was no difference by the grade of atypia (Table 2).

### Adenoma Atypia and Morphology

All lesions showing mild atypia were classified as IIa. Although the majority of lesions showing moderate and severe atypia were similarly IIa, depressed type lesions were more prevalent in cases of severe atypia (9 of 25 lesions, 31.0%) than in cases of moderate atypia (18 of 119 lesions, 15.1%) (Table 3).

**Table 1** Adenoma Atypia and Size

| | | *Size (mm)* |
|---|---|---|
| Mild atypia | ($n$ = 9) | 5.1 ± 1.9 |
| Moderate atypia | ($n$ = 119) | 4.9 ± 2.0 |
| Severe atypia | ($n$ = 25) | 5.5 ± 2.3 |

**Table 2** Adenoma Atypia and Site

| | | *R* | *S* | *D* | *T* | *A* | *C* |
|---|---|---|---|---|---|---|---|
| Mild atypia | ($n$ = 9) | 0 | 5 | 1 | 3 | 0 | 0 |
| Moderate atypia | ($n$ = 119) | 4 | 41 | 30 | 33 | 8 | 3 |
| Severe atypia | ($n$ = 25) | 2 | 9 | 3 | 11 | 0 | 0 |

Abbreviations: R, rectum; S, sigmoid colon; D, descending colon, T, transverse colon; A, ascending colon; C, cecum.

**Table 3** Adenoma Atypia and Macroscopic Classification

| | | *IIa* | *IIaIIc* | *IIb* | *IIc* |
|---|---|---|---|---|---|
| Mild atypia | ($n$ = 9) | 9 | 0 | 0 | 0 |
| Moderate atypia | ($n$ = 119) | 98 | 15 | 3 | 3 |
| Severe atypia | ($n$ = 25) | 16 | 8 | 0 | 1 |

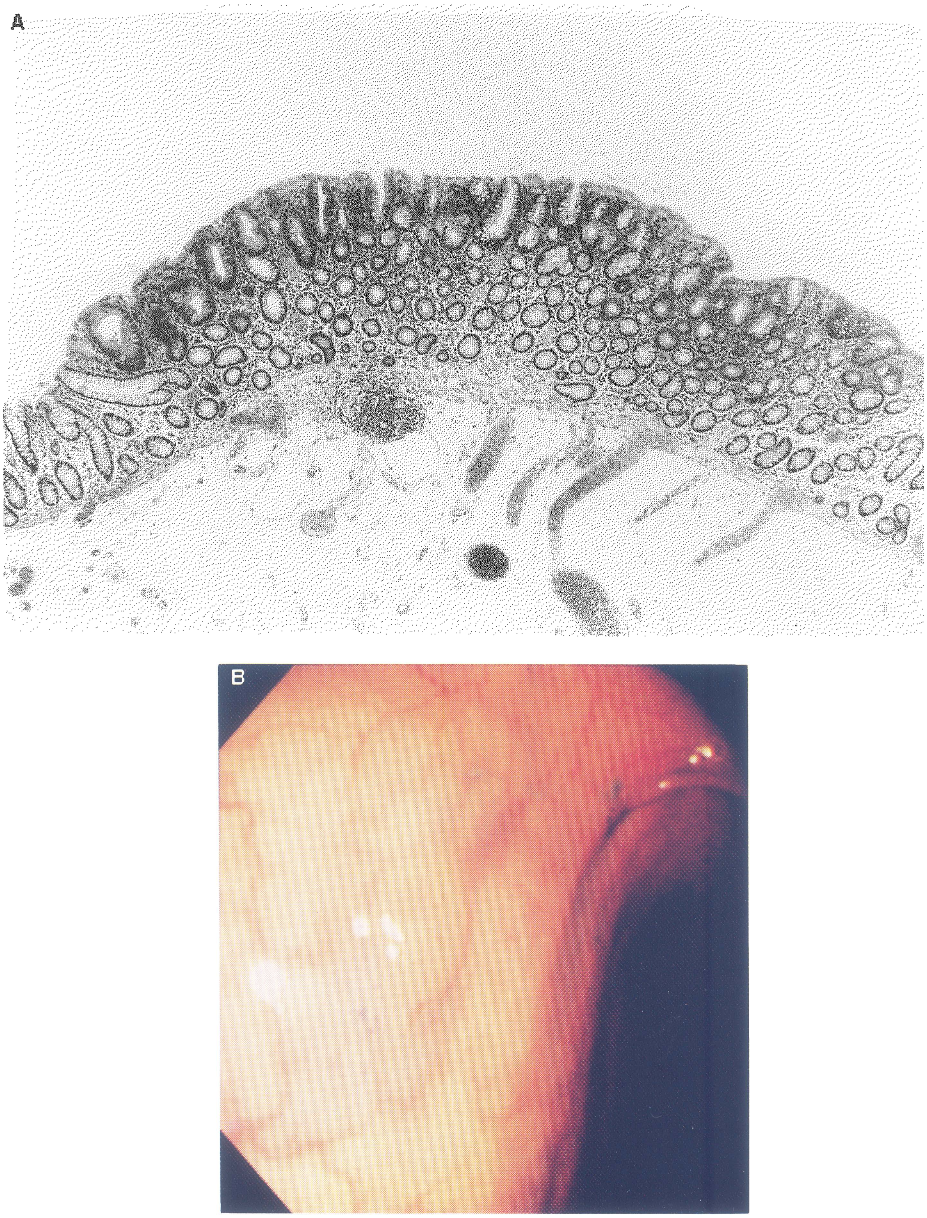

**Figure 1** A. Microscopic view of an $m_1$ lesion, showing invasion of only the surface layer of the mucosa (HE stain, ×10). B. Endoscopic view of the same $m_1$ lesion.

### Adenoma Atypia and Location in the Mucosa

Location in the mucosa was $m_1$ for 5 lesions, $m_2$ for 2 lesions, and $m_3$ for 2 lesions in the mild atypia group, $m_1$ for 52 lesions, $m_2$ for 34 lesions, and $m_3$ for 33 lesions in the moderate atypia group, and $m_1$ for 8 lesions, $m_2$ for 7 lesions, and $m_3$ for 10 lesions in the severe atypia group. Higher grades of atypia were associated with greater proportions of $m_3$ lesions (Table 4).

### Localization in the Mucosa and Macroscopic Classification

Analysis of the relationship between the location in the mucosa and macroscopic type revealed IIa lesions to be the most common lesions in the $m_1$, $m_2$, and $m_3$ groups. In the $m_3$ group, there was a relatively high proportion of IIaIIc and IIc lesions (Table 5). After dividing the lesions into depressed type lesions (IIaIIc and IIc) and nonde-

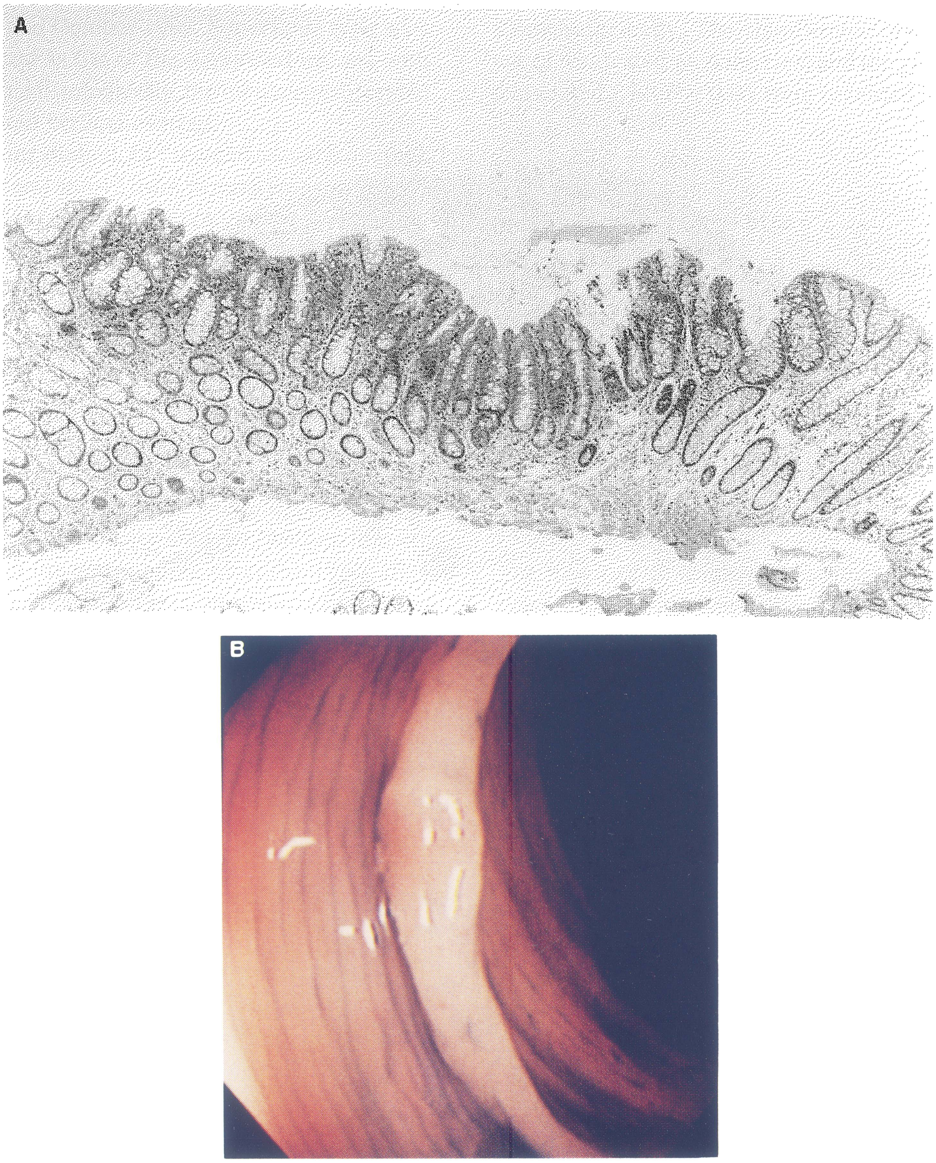

**Figure 2** A. Microsopic view of an $m_2$ lesion. Invasion is deeper than an $m_1$ lesion but it does not reach the muscularis mucosae (HE stain, ×13.2). B. Endoscopic view of the same $m_2$ lesion.

**Table 4** Adenoma Atypia and Location in the Mucosa

| | | $m_1$ | $m_2$ | $m_3$ |
|---|---|---|---|---|
| Mild atypia | ($n$ = 9) | 5 | 2 | 2 |
| Moderate atypia | ($n$ = 119) | 52 | 34 | 33 |
| Severe atypia | ($n$ = 25) | 8 | 7 | 10 |

**Table 5** Location in the Mucosa and Macroscopic Classification

| | | *IIa* | *IIaIIc* | *IIb* | *IIc* |
|---|---|---|---|---|---|
| $m_1$ | ($n$ = 65) | 60 | 3 | 1 | 1 |
| $m_2$ | ($n$ = 43) | 33 | 9 | 1 | 0 |
| $m_3$ | ($n$ = 45) | 30 | 11 | 1 | 3 |

pressed type lesions (IIa and IIb), significant differences were noted between the $m_1$ group and the $m_2$ group ($P < 0.05$), and between the $m_1$ group and the $m_3$ group ($P < 0.01$) (Table 6).

### Adenoma Atypia and Height

Although there was no difference by the thickness of the surrounding mucosa, lesion height tended to be lower with higher grades of atypia (Table 7).

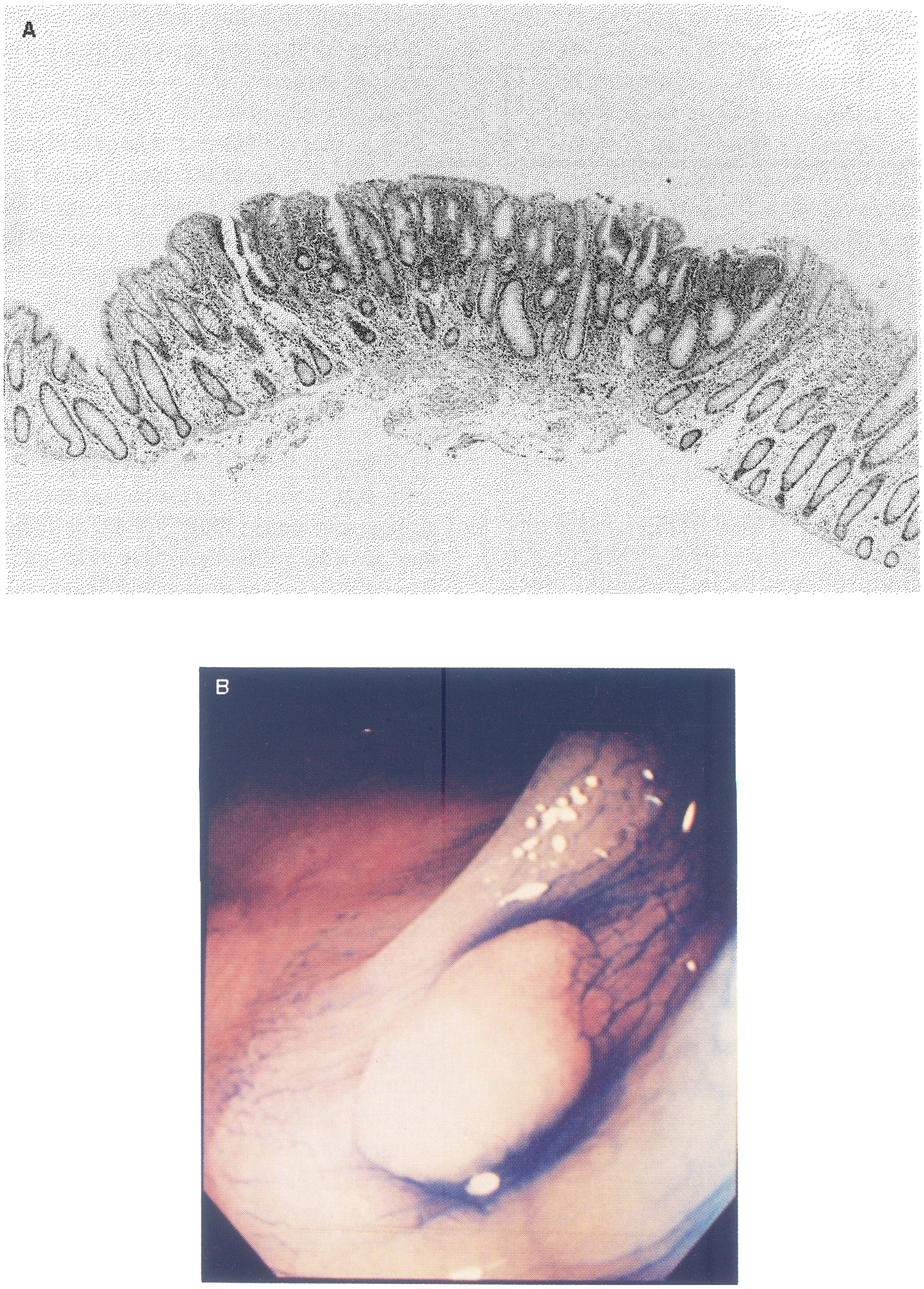

**Figure 3** A. Microscopic view of an $m_3$ lesion. Invasion extends to the muscularis mucosae (HE stain, ×10). B. Endoscopic view of the same $m_3$ lesion.

**Table 6** Location in the Mucosa and Macroscopic Depression

| | | *Depressed type* | *Nondepressed type* |
|---|---|---|---|
| $m_1$ | ($n$ = 65) | 4 (6.1%) | 61* |
| $m_2$ | ($n$ = 43) | 9 (20.9%) | 34† |
| $m_3$ | ($n$ = 45) | 14 (31.1%) | 31 |

*$P < 0.05$.
†$P < 0.01$.

**Table 7** Adenoma Atypia and Height

| | | *Thickness of the surrounding mucosa (μm)* | *Lesion height (μm)* |
|---|---|---|---|
| Mild atypia | ($n$ = 9) | 511 ± 109 | 394 ± 189 |
| Moderate atypia | ($n$ = 119) | 518 ± 90 | 367 ± 243 |
| Severe atypia | ($n$ = 25) | 572 ± 114 | 314 ± 250 |

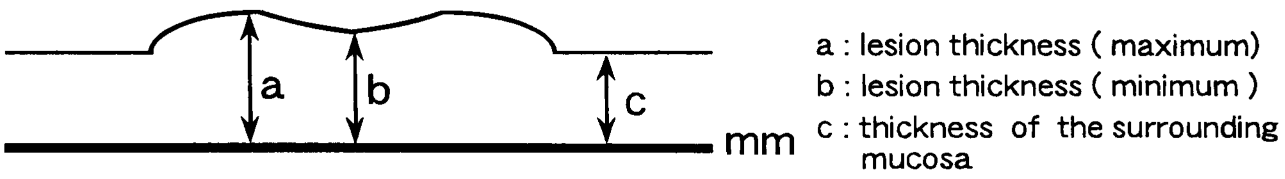

$$\text{Lesion height} = \frac{a+b}{2} - c$$

**Figure 4** Method used for calculating lesion height.

### Location in the Mucosa and Height

Analysis of the relationship between location in the mucosa and lesion height indicated that adenoma height was greatest in the $m_1$ group followed by, in descending order, the $m_2$ and $m_3$ groups. The difference in adenoma height between the $m_1$ group and $m_3$ group was significant (Table 8).

**Table 8** Location in the Mucosa and Height

| | | *Thickness of the surrounding mucosa (μm)* | *Lesion height (μm)* |
|---|---|---|---|
| $m_1$ | ($n = 65$) | 533 ± 107 | 413 ± 298 |
| $m_2$ | ($n = 43$) | 516 ± 88 | 346 ± 152* |
| $m_3$ | ($n = 45$) | 526 ± 92 | 295 ± 200 |

*$P < 0.05$.

### DISCUSSION

Adenomas more than 1 cm in diameter were previously regarded to have a high clinical risk of progression to carcinoma. Recently, however, the existence of superficial type adenomas has attracted considerable interest (5–7), making the delineation of their morphological features an urgent task. As many superficial type adenomas are depressed, these lesions, similar to pedunculated adenomas, cannot be readily distinguished from superficial type colonic carcinomas based on size alone. We therefore studied a series of endoscopically resected superficial type adenomas to better understand their morphological characteristics.

The grade of atypia, classified as mild, moderate, or severe, was unrelated to lesion size, which ranged from 4.9 to 5.5 mm. Although larger lesions have been associated with higher frequencies of severe atypia and carcinogenesis in pedunculated adenomas, this does not appear to be true for superficial type adenomas.

The relationship between the lesion site in the colon and the degree of atypia was also studied. Superficial type adenomas were situated primarily from the sigmoid colon to the transverse colon, and the lesion site was unrelated to the grade of atypia. Overall trends approximated those for pedunculated adenomas, but relatively few superficial type adenomas were found in the rectum, compared to trends for colonic polyps as a whole. This was attributed to the relatively high number of hyperplastic polyps found in the rectum (total rectal polyps: 1,060), compared to other segments of the colon (total colonic polyps: 6,209) at our institute (8).

Depression has been reported to be one of the morphological characteristics of superficial type colonic carcinomas. In the present study, depressed type was seen in 18 of 119 lesions (15.1%) with moderate atypia and in 9 of 25 lesions (31.0%) with severe atypia. Although our series included adenomas only, the number of depressed type lesions was higher with higher grades of atypia. Other investigators have similarly found that depressed type lesions are not necessarily carcinomas, and that, on the contrary, most are adenomas. In one study, 93.1% of lesions with relative depression of ≤5 mm were reported to be adenomas (9). By applying the criteria of Ajioka et al (9). to define absolute depression and relative depression, only 1 of the 153 adenomas in our series (3.9%) showed absolute depression. Depression associated with superficial type adenomas therefore appears to be relative depression in the overwhelming majority of cases. Therefore, moderately and severely atypical lesions tend to be depressed, which makes it more difficult to differentiate these lesions from carcinomas.

Since location in the mucosa is considered to closely reflect macroscopic lesion morphology, this factor was also studied for our series of superficial type adenomas. Location of tubules was classified as $m_1$, $m_2$, or $m_3$. Lesions with mild atypia were mostly $m_1$, but there were relatively more $m_2$ and $m_3$ lesions among the moderate and severe atypia groups. This may be related to the growth and development of tubules, i.e., higher grades of atypia were apparently associated with deeper invasion of the mucosa. Thus, morphologically, $m_2$ and $m_3$ adenomas tended to be IIaIIc or IIc depressed type lesions. Depression was shown by 9 of 43 lesions (20.9%) in the $m_2$ group and 14 of 30 lesions (31.1%) in the $m_3$ group. These rates were significantly higher than in the $m_1$ group (4 of 65 lesions, 6.1%). Therefore, lesions showing $m_2$ and $m_3$ patterns in the mucosa were associated with depression morphologically and moderate or severe atypia histologically.

The height of superficial type adenomas was also compared. During endoscopic examination, lesion height may vary considerably depending on the degree of insufflation. In histopathological specimens height may be affected by how resected materials are distended before formalin fixation (10). To enhance accuracy, we therefore measured the lesion thickness and the thickness of the surrounding mucosa. The thickness of the surrounding mucosa was subtracted from adenoma thickness to derive lesion height.

The thickness of the surrounding mucosa ranged from 511 to 572 μm and was unrelated to the grade of atypia. Severely atypical adenomas were not as high as moderately and mildly atypical adenomas, but the differences were not significant. However, analysis of the relationship between location in the mucosa and adenoma height revealed a significant difference in height between the $m_1$ group and the $m_3$ group, indicating that location was more closely associated with local morphology than was atypia. Since many $m_2$ and $m_3$ lesions are depressed, it can be assumed that the mean height of these lesions decreases. Tsuda et al. (10) reported lesion height to be 304 to 568 μm for superficial protruding type lesions without depression, compared with only 174 to 203 μm for those with depression. We found lesion height to be $390 \pm 237$ μm for adenomas without depression and $220 \pm 213$ μm for lesions with depression, in general agreement with the result of Tsuda et al.

### CONCLUSIONS

The following conclusions were obtained from clinical and histological observations of endoscopically resected superficial type adenomas:

1. There was no relationship between the grade of atypia and size.
2. Morphological characteristics (depression, etc.) were more closely associated with location in the mucosa than with the grade of atypia.
3. By classifying location of the tubules as $m_1$, $m_2$, and $m_3$, it was found that the $m_2$ and $m_3$ groups included significantly more depressed type lesions than did the $m_1$ group.
4. Adenoma height ranged from 295 to 413 μm; height was lowest in the $m_3$ group followed by, in ascending order, the $m_2$ and $m_1$ groups.

### ACKNOWLEDGMENT

The authors would like to express their appreciation to Peter Star of Medical Network K.K. for translating the manuscript for this paper.

### REFERENCES

1. Castleman B, Krickstein HI. Do adenomatous polyps of the colon become malignant? N Engl J Med 1962;267:469–475.
2. Morson BC. Precancerous and early malignant lesions of the large intestine. Br J Surg 1968;55:725–731.
3. Nakamura K, Shibuya S, Nishizawa M, et al. Adenoma-carcinoma sequence of colorectal carcinomas analyzed by the use of objective indices of grade of atypicality, and their growing processes in early phase. Stomach Intest 1985;20:877–888.
4. Kudo S, Muto T, Soga J, et al. Development process of colorectal adenoma and early carcinoma. Stomach Intest 1985;20:903–910.
5. Hasegawa K, Mikami T, Noguchi T, et al. Colonoscopic diagnosis of flat tumors. Gastroenterol Endosc 1984;26:1692–1699.
6. Muto T, Kamiya J, Sawada T, et al. Small "flat adenoma" of the large bowel with special reference to its clinicopathologic features. Dis Colon Rectum 1985;28:847–851.
7. Matsukawa M, Neki T, Usui Y, et al. Macroscopic features and anatomic distribution of colorectal adenoma. Stomach Intest 1989;24:155–160.
8. Fujinuma S, Sakai Y, et al. Clinical features of superficial type colonic tumors. Tokyo: Igakusha 1994:135–141.
9. Ajioka Y, Watanabe H, Honma T, et al. Problems in biopsy and diagnosis of depressed type early carcinomas of the colon. Clinic Gastroenterol 1992;7:393–403.
10. Tsuda S, Yao T, Matsui T, et al. Superficial colonic neoplasm diagnosed by endoscopy: comparison of radiologic and morphologic pictures. Stomach Intest 1992;27:935–947.

